# Molecular docking analysis of bioactive compounds from *Cissampelos pareira* with PPAR gamma

**DOI:** 10.6026/97320630018265

**Published:** 2022-03-31

**Authors:** Umapathy Vidhya Rekha, Ponnulakshmi Rajagopal, Rama Varadhachariyar, Puja Harie Priya, JH Shazia Fathima, Ramajayam Govindan, ChellaPerumal Palanisamy, KB Aswini Brindha, Vishnu Priya Veeraraghavan, Selvaraj Jayaraman

**Affiliations:** 1 Department of Public Health Dentistry, Sree Balaji Dental College and Hospital, Pallikaranai, Chennai-600 100, India; 2 Central Research Laboratory, Meenakshi Academy of Higher Education and Research (Deemed to be University), Chennai, India; 3 Department of Biochemistry, Sri venkateshwara Dental College and Hospital, Thalambur, Chennai, India; 4 Department of Prosthodontics, Ragas Dental College and Hospital, Uthandi, Chennai, India; 5 Department of Oral and Maxillofacial Pathology, Ragas Dental College and Hospitals, Chennai, India; 6 Multi Disciplinary Research Unit, Madurai Medical College, TamilNadu, India; 7 State Key Laboratory of Biobased Materials and Green Papermaking, College of Food Science and Engineering, Qilu University of Technology, Shandong Academy of Science, Jinan 250353, China; 8 Department of Biochemistry, Asan Memorial Dental College and Hospital, Tamil Nadu, India; 9 Department of Biochemistry, Saveetha Dental College and Hospitals, Saveetha Institute of Medical and Technical Sciences, Saveetha University, Chennai, India

**Keywords:** Cissampelospareira, PPARγ, diabetes mellitus, molecular docking

## Abstract

We document the Molecular docking analysis of bioactive compounds from *Cissampelos pareira* with PPAR gamma for further consideration in drug discovery for T2DM.

## Background:

Diabetes mellitus caused by a deficiency in insulin secretion or function, or both, and results in tissue and vascular damage, as well as a variation of complications [1,2]. Type 2 diabetes (T2D) is a chronic disorder of food metabolism induced by decreased insulin action [3]. General practitioners often prescribe a mixture of antidiabetic agents for T2D treatment, and an excess of antidiabetic medications may result in extreme hypoglycemia, which can have severe toxic and adverse effects. This prompted the scientific community to conduct research on novel anti-diabetic agents [4]. Numerous plants and plant sections have been identified as having antidiabetic properties. Numerous plant-derived active principles describing a variety of bioactive compounds have been demonstrated to be useful for potential use in T2D therapeutics [5].Identifying the optimal goal for diabetes management has been a focus of study in recent years. The peroxisome proliferator-activated receptor gamma (PPAR, PDB: 2PRG) transcription factor is a member of the nuclear receptor super family and is a critical regulator of target genes involved in glucose and lipid homeostasis [6]. PPAR and its targets have been identified as promising therapeutic targets for type 2 diabetes [7, 8].With the rapid advancement of biological and chemical knowledge, docking has reshaped research and expanded avenues for drug applicant identification. Molecular docking is an effective method for discovering new small molecule drugs that target proteins [9]. Therefore, it is of interest to document the Molecular docking analysis of bioactive compounds from *Cissampelos pareira* with PPAR for further consideration in drug discovery for T2DM.

## Materials and Methods:

### Retrieval of the Three-Dimensional structure of target protein:

The structures of the target human peroxisome proliferator-activated receptor gamma (PDB: 2PRG)[10] was downloaded from RCSB protein Data Bank, and prepared for molecular docking simulation in just such a way that all heteroatomes (i.e. non-receptor atoms such as water, ions, etc.) were removed.

### Ligand Preparation:

Ten compounds from *Cissampelos pareira* that are collected from the literature (Table 1 - see PDF). The structures of all these compounds have also been obtained from the PubChem Compound Database in the Spatial Data File (.SDF) format and translated to the PDB file format by using Online Smile Translator. Energy minimization of ligands has been completed using Open Babel software with a steepest descent using uniform force fields and then translated to PDBQT format.

### Molecular docking:

AutoDock (V. 4.0) could be used in the PyRx GUI to validate the binding capability of the selected ligands to the entire selected target [12]. Docking offers a valuable view of the interactions between ligand proteins. During the docking period, ligands were considered to be flexible and the protein was assumed to be rigid. The grid configuration report was created using the Pyrex Auto Grid software. Execution was also used to know/predict amino acids that come into contact with ligands at the active protein site. Results less than 1.0Å in the position root-mean-quarter deviation (RMSD) were considered to be optimal and grouped together to find an acceptable binding. The highest binding energy (most negative) has been identified to be a ligand with high binding affinity. The docking poses collected at each compound have been rated according to their dock score feature and the best docking result was further analysed using Pymol.

## Results and Discussion:

Numerous medicinal plants contain phytochemicals that have been confirmed to have anti-diabetic properties. These phytochemicals are frequently used to treat or alleviate the symptoms of diabetes. The present study examined ten compounds derived from *Cissampelos pareira* and their active anti-diabetic action. The compound was docked with the human protein PPARγ. The best confirmation for each compound was chosen based on its binding affinity. The active conformations of the compounds from the selected data have been described as well as their interactions with the target protein. Compounds were chosen for their low binding energy to the protein pocket. The interactions have been detected by checking all amino acids in the target's active site and the atoms in the compounds one by one. The binding interactions have been mediated by the amino acids contained inside the active site pocket. The Auto dock PyRx software was used to simulate docking in the active sites of PPARγ. This program has been shown to successfully replicate experimentally observed binding modes in terms of minimum docking energy. The best possible binding modes of all the ligands at target protein active sites were displayed in Figure 1(see PDF) by using PYMOL tool v1.1. Ligands hydrogen-bonding to four compounds and their corresponding energy values are listed in Table 2 & Figure 1a(see PDF) depicts the docking analysis of PPARγ R trans-N-feruloyltyramine. These results showed the hydrogen-bonding network of these compounds formed the interaction with conserved PPARγ residues. This compound formed two hydrogen bond interactions through the amino acids residues of GLN-294, SER-342. Figure 1(b)(see PDF) showed the hydrogen-bonding interaction between the (-)-cyclanoline with PPARγ. It showed effective binding in terms of lowest binding score of -6.8kcal/mol. And also it formed the two hydrogen bond interaction with ARG-228 and GLU-343. Figure 1c(see PDF) depicted the orientation of curine bound in the active site of the PPARγ crystal structure. Figure 1d(see PDF) illustrated hydrogen-bonding interaction of (+)-coclaurine to PPARγ protein. The selected four compounds exhibited good hydrogen-bond interaction with PPARγ. Therefore, these compounds may act against PPARγ and potential lead for diabetes management.

## Conclusion:

We show the molecular docking analysis data of Trans-N-feruloyltyramine, ()-cyclanoline, curine, and (+)-coclaurine *Cissampelos pareira* with PPAR gamma for further consideration in drug discovery and development for T2DM.
